# U.S. medical resident familiarity with national tuberculosis guidelines

**DOI:** 10.1186/1471-2334-7-89

**Published:** 2007-08-02

**Authors:** Petros C Karakousis, Frangiscos G Sifakis, Ruben Montes de Oca, Valerianna C Amorosa, Kathleen R Page, Yukari C Manabe, James D Campbell

**Affiliations:** 1 Department of Medicine, Johns Hopkins University School of Medicine, Baltimore, USA; 2 Department of International Health, Johns Hopkins Bloomberg School of Public Health, Baltimore, USA; 3 Department of Epidemiology, Johns Hopkins Bloomberg School of Public Health, Baltimore, USA; 4 Department of Medicine, University of Pennsylvania School of Medicine, Philadelphia, USA; 5 Department of Pediatrics, University of Maryland School of Medicine, Baltimore, USA

## Abstract

**Background:**

The ability of medical residents training at U.S. urban medical centers to diagnose and manage tuberculosis cases has important public health implications. We assessed medical resident knowledge about tuberculosis diagnosis and early management based on American Thoracic Society guidelines.

**Methods:**

A 20-question tuberculosis knowledge survey was administered to 131 medical residents during a single routinely scheduled teaching conference at four different urban medical centers in Baltimore and Philadelphia. Survey questions were divided into 5 different subject categories. Data was collected pertaining to institution, year of residency training, and self-reported number of patients managed for tuberculosis within the previous year. The Kruskal-Wallis test was used to detect differences in median percent of questions answered correctly based on these variables.

**Results:**

The median percent of survey questions answered correctly for all participating residents was 55%. Medical resident knowledge about tuberculosis did not improve with increasing post-graduate year of training or greater number of patients managed for tuberculosis within the previous year. Common areas of knowledge deficiency included the diagnosis and management of latent tuberculosis infection (median percent correct, 40.7%), as well as the interpretation of negative acid-fast sputum smear samples.

**Conclusion:**

Many medical residents lack adequate knowledge of recommended guidelines for the management of tuberculosis. Since experience during training influences future practice pattterns, education of medical residents on guidelines for detection and early management of tuberculosis may be important for future improvements in national tuberculosis control strategies.

## Background

Although the overwhelming global burden of *Mycobacterium tuberculosis *disease occurs in developing countries, developed countries have not been able to eradicate this infection. After an increase in tuberculosis cases from 1985–1992, implementation of effective interventions at the national, state, and local levels led to a decline in the incidence of tuberculosis in the U.S. by 44% from 1993 to 2003 [1], to a historic low level (14,874 cases, representing 5.1 cases/100,000 population in 2003) [2]. However, as a result of continued immigration from countries with a high prevalence of tuberculosis [3], it is possible that recent encouraging trends in tuberculosis incidence in the U.S. may be reversed in the near future. In addition, the estimated 10 to 15 million persons in the United States with latent tuberculosis infection [1] represent a significant reservoir for subsequent tuberculosis reactivation.

Social factors, including homelessness, injection drug use, incarceration, and immigration, as well as concurrent HIV infection, have contributed to the high prevalence of tuberculosis in urban areas in the US [4]. Many of these patients will be seen and evaluated first by internists and primary care physicians. Because care patterns learned in training are reflected in later practice [5], it is important to ensure that future internists have an appropriate awareness of this important infection. From a public health perspective, medical resident physicians training at urban medical centers are likely to be present when patients with previously undiagnosed tuberculosis first encounter the medical health system.

We evaluated medical resident knowledge of tuberculosis diagnosis and early management based on American Thoracic Society guidelines [6-10] at four urban internal medicine residency training programs in Baltimore and Philadelphia. We assessed knowledge based on a brief written examination and then evaluated scores on the examination with respect to institution, post-graduate year of training, and number of tuberculosis cases seen within the past year. As a result, we identified specific areas of knowledge deficiency that we believe require further attention in medical residency curricula.

## Methods

A survey (see Appendix 1) comprising 20 questions (11 multiple-choice and 9 true/false) was developed by the collaborating authors and reviewed for face and content validity. The survey was pilot tested among 17 infectious disease attending physicians from the participating institutions, 8 of whom described themselves as tuberculosis experts. The median percent of survey questions answered correctly in this group was 90%, and based on their responses, a single question (Question # 2) was deemed ambiguous by the participating authors, and this question was omitted from the data analysis. The knowledge surveys were administered anonymously to internal medicine residents at the following four accredited U.S. residency training programs: Johns Hopkins Hospital, Baltimore, MD; Johns Hopkins Bayview Medical Center, Baltimore, MD; University of Maryland Medical Center, Baltimore, MD; and Hospital of the University of Pennsylvania, Philadelphia, PA. The collaborating authors administered surveys during a single routinely scheduled teaching conference at each of their respective institutions in the final month of the academic year in 2005. To prevent residents from studying the subject in anticipation, the surveys were distributed without advance notice. Medical residents were allowed up to 30 minutes to complete the surveys without outside assistance. Correct responses were determined prior to survey administration according to published American Thoracic Society guidelines (see Appendix 2) [6-10]. Each completed survey was assigned a score based on the percent of questions answered correctly, with a maximum survey score of 100% representing correct responses to all 19 questions.

Data were collected pertaining to institution, year of residency training, and self-reported number of patients managed for tuberculosis within the previous year. For purposes of data analysis, each institution randomly was assigned a number from 1 to 4. We used the Kruskal-Wallis test to detect differences in median survey scores based on these variables. Survey questions were divided into 5 categories: transmission of *M. tuberculosis *(questions #3, 12, and 13); diagnosis and management of latent tuberculosis infection (questions #1, 4, 5, 6, 7, 10, and 11); diagnosis of active tuberculosis (questions #14, 15, 16, 17, and 18); toxicity of anti-tuberculosis therapy (questions #8, 9, and 19); and HIV and tuberculosis co-infection (questions #7, 19, and 20).

This study was approved by the Institutional Review Board of each participating institution, and the need for written informed consent was waived.

## Results

### General characteristics of survey respondents

The surveys were completed by 131 medical residents, representing 29% of the 446 total medical residents training at the four participating institutions. Survey response rates among conference attendees at each institution were 100%. Forty-one percent (n = 53) of participating residents were from institution 1, 21% (n = 28) were from institution 2, 16% (n = 21) were from institution 3, and 22% (n = 29) were from institution 4. Respondents were evenly distributed with respect to their level of residency training; 35% (n = 46) were post-graduate year (PGY)-1, 32% (n = 41) were PGY-2, and 33% (n = 43) were PGY-3. Nearly half of all respondents (48%, n = 60) had directly cared for only 1 or 2 patients with tuberculosis in the previous year, and 29% (n = 36) of respondents had cared for 3 or more patients with tuberculosis. Twenty-three percent (n = 29) had not cared for a single patient with tuberculosis in the previous year (Table 1).

**Table 1:** Medical Resident Background and Median Survey Scores (Percent Correct)

	**N (%)**	**Median score**
**Overall**	131 (100)	55.0

**Institution**		
**1**	53 (40.5)	55.0
**2**	28 (21.4)	55.0
**3**	21 (16.0)	50.0
**4**	29 (22.1)	65.0
**p-value**		0.03

**TB patients seen within last year**		
**0**	29 (23.2)	55.0
**1–2**	60 (48.0)	60.0
**3 or more**	36 (28.8)	52.5
**p-value**		0.04

**Years of training**		
**1**	46 (35.4)	55.0
**2**	41 (31.5)	55.0
**3**	43 (33.1)	55.0
**p-value**		0.80

The median survey score for all participating medical residents was 55%. Overall, survey performance did not correlate with level of residency training, as median scores for PGY-1, PGY-2, and PGY-3 residents were 55% for all years (p = 0.94). Interestingly, median scores among residents who had directly cared for 3 or more patients with tuberculosis within the previous year (52%) were significantly lower than those among residents who had cared 1–2 patients (60%; p = 0.04). Finally, survey scores were somewhat influenced by institution, as median scores for institutions 1, 2, 3, and 4 were 55%, 55%, 50%, and 65%, respectively (p = 0.03) (Table 1).

### Transmission of *Mycobacterium tuberculosis*

The median score for all medical residents in the category of *M. tuberculosis *transmission was 95% (Table 2). Half of all medical residents (n = 66) responded correctly to all 3 relevant questions. Eighty-one percent (n = 106) recognized correctly that no specific effective measures exist to prevent acquisition of *M. tuberculosis *in areas of high prevalence of the disease, and only 5% (n = 7) incorrectly recommended vaccination with BCG for a traveler to a tuberculosis endemic area (Question #3). Eighty-seven percent of residents (n = 112) correctly identified patients with extrapulmonary tuberculosis as being less likely to transmit *M. tuberculosis *than those with pulmonary involvement (Question #13). Seventy-two percent of residents (n = 36) correctly recognized that patients whose sputum samples are negative by acid fast staining may still transmit *M. tuberculosis *(Question #12).

**Table 2:** Median percent of questions answered correctly by all residents in each subject category

**Subject category**	**Percent correct (median)**
Transmission of *M. tuberculosis*	95.0
Diagnosis and management of latent tuberculosis infection	40.7
Diagnosis of active tuberculosis	57.0
Toxicity of therapy for active tuberculosis	63.3
Human immunodeficiency virus and tuberculosis co-infection	63.3

### Diagnosis and management of latent tuberculosis infection

The median score for all medical residents in the category of latent tuberculosis infection was 40.7%. Although medical residents from institution 4 received the highest median scores in this category (median, 54.2%, p < 0.01), their scores represent only one more correct answer above median scores from all institutions combined. Interpretation of tuberculin skin test results posed particular difficulty for the participating medical residents. When presented with a clinical vignette describing a foreign-born, BCG-vaccinated individual, whose tuberculin skin test is positive (Question #1), fewer than half of residents would treat with the appropriate regimen of isoniazid for 9 months (47%; n = 62). Among those responding incorrectly to this question, 71% believed that the positive tuberculin skin test was attributable to prior BCG vaccination, and recommended no specific therapy for latent tuberculosis infection. Sixty-six percent of residents (n = 85) correctly recognized that a tuberculin skin test inducing a 5-mm reaction is not considered positive for health care workers (Question #5).

With respect to the therapy of latent tuberculosis infection, only 41% of residents (n = 53) identified the combination of rifampin and pyrazinamide as an unacceptable regimen, and half of residents (n = 67) were aware of the acceptability of a 4-month regimen of rifampin for the treatment of latent tuberculosis infection (Question #4). Only 15% (n = 108) were aware of the proper use of pyridoxine supplementation (Question #10), with three-quarters of incorrect responders choosing not to recommend pyridoxine supplementation during pregnancy.

Nearly half of residents (n = 64) believed incorrectly that the lifetime risk of developing active disease in non-HIV-infected persons with latent tuberculosis infection was below 2 percent (Question #6). Fifty-five percent of residents (n = 71) correctly identified the use of infliximab as the greatest risk factor for progression from latent tuberculosis infection to active disease among other possible risk factors including age, rheumatoid arthritis, methotrexate use, and smoking history (Question #11).

### Diagnosis of active tuberculosis

The median score for all medical residents in the category of active tuberculosis diagnosis was 57%. The great majority of residents (97 percent, n = 125) correctly indicated that the diagnosis of pulmonary tuberculosis cannot be reliably excluded on the basis of a negative tuberculin skin test (Question #14). However, only 28% of residents (n = 93) recognized that the diagnosis of pulmonary tuberculosis cannot be reliably excluded after 3 sequential sputum samples are negative by acid fast staining (Question #15). Similarly, only 40% of residents (n = 78) recognized that the diagnosis of pulmonary tuberculosis cannot be reliably excluded after a single bronchoscopic alveolar lavage sample is negative by acid fast staining (Question #16). Regarding the use of nucleic acid amplification testing of sputum samples in the diagnosis of pulmonary tuberculosis, roughly three-quarters of residents (n = 101) recognized the high specificity of the test for acid fast-positive sputum samples (Question #17), and two-thirds (n = 89) recognized the reduced sensitivity of the test for acid-fast sputum negative samples (Question #18).

### Toxicity of therapy for active tuberculosis

The median score for all medical residents in the category of anti-tuberculosis therapy-associated toxicity was 63.3%. With respect to initial four-drug combination therapy for active tuberculosis cases, 80% of residents were aware of rifampin-related staining of secretions, and 90% were aware of the potential hepatotoxicity of the regimen (Question #8). When confronted with a patient experiencing a 2-fold elevation in aspartate aminotransferase (AST) above baseline values after initiation of therapy for active tuberculosis, 30% of residents (n = 39) incorrectly recommended discontinuation of one or more anti-tuberculous drugs (Question #9).

### Human immunodeficiency virus and tuberculosis co-infection

The median score for all medical residents in the category of HIV and tuberculosis co-infection was 63.3%. Many residents (39%, n = 51) incorrectly thought that the annual risk of developing active disease in HIV-infected patients with latent tuberculosis infection was less than or equal to 1 percent (Question #7).

The majority of residents (84%, n = 102) correctly identified rifampin as the first-line anti-tuberculous agent with the greatest potential for drug-drug interactions in patients receiving highly active antiretroviral therapy (HAART) (Question #19). Nearly 80% of residents (n = 105) correctly would not delay initiation of HAART until active tuberculosis had been completely treated in HIV-infected patients with CD4 cell counts below 100 cells/mm^3 ^(Question #20).

## Discussion

One major challenge facing current tuberculosis eradication efforts in the U.S. is the maintenance of clinical and public health expertise in an era of declining disease incidence. Lack of such expertise may lead to delayed detection of active tuberculosis cases, resulting in delayed initiation of treatment and the potential for continued transmission of the organism [11-15]. From a public health point of view, it is imperative that medical housestaff training at urban medical centers be able to recognize and promptly treat this infection, since these centers treat the majority of tuberculosis cases in the U.S. [16,17]. It is also important to ensure that future internists are competent in the diagnosis and early management of latent and active tuberculosis infections.

Consistent with previous reports indicating defiencies in U.S. physician trainee knowledge of national disease-specific guidelines [18-20], our study revealed that many medical residents lack adequate knowledge of recommended guidelines for the management of tuberculosis. Contrary to our initial hypotheses, medical resident knowledge about tuberculosis did not improve with increasing PGY level of training or volume of tuberculosis patients. Interestingly, the lowest median scores were observed in the group of residents who had directly cared for the greatest number of patients with tuberculosis within the previous year. This finding, although statistically significant, may not be clinically relevant, since median scores between the groups differed at most by a single question. Alternatively, medical residents who had directly cared for a large number of patients with tuberculosis within the previous year may have done so during rotations in developing countries, where tuberculosis education may not comply with current U.S. guidelines. In support of this hypothesis, a recent study demonstrated poor awareness of and low compliance with published tuberculosis guidelines among medical interns in Pakistan, a country with a high prevalence of tuberculosis [21]. Although resident median scores were somewhat influenced by institution, the median score at the institution with the highest scores exceeded the overall median by only a single correct answer.

In order to gain insight into specific areas of resident knowledge deficiency, we divided the questions into 5 specific categories (see Methods). Among these categories, the median score was highest for questions pertaining to transmission of *M. tuberculosis *(95.0%), with nearly half of all residents responding correctly to all questions within this category. The category for which the medical residents received the lowest median score was that of diagnosis and management of latent tuberculosis infection (median score, 40.7%). This result is perhaps not surprising, since internal medicine residency training in the U.S. occurs mostly in the inpatient setting [22], while latent tuberculosis infection is often diagnosed and managed in the outpatient setting.

Analysis of individual questions pertaining to latent tuberculosis infection revealed several common misconceptions among medical residents. One common error was the false attribution of a positive tuberculin skin test to distant BCG vaccination in a person from a tuberculosis-endemic country. Although prior receipt of BCG vaccine in some cases may lead to a positive tuberculin skin test, this effect wanes significantly over time [23-25]. Therefore, current guidelines recommend treatment for latent tuberculosis infection in persons with positive skin tests who are from tuberculosis-endemic areas, regardless of prior BCG vaccination history [8]. Recently developed interferon gamma release assays using *Mycobacterium tuberculosis*-specific antigens have shown promise in diagnosing latent tuberculosis infection in high-risk individuals [26,27], and appear to have the advantage of decreased cross-reactivity in individuals exposed to BCG and nontuberculous mycobacteria [28]. A large proportion of the residents was not familiar with appropriate and inappropriate treatment regimens for latent tuberculosis infection, with less than half of residents identifying 2 months of rifampin and pyrazinamide as unacceptable, despite the proscription of this regimen in current guidelines due to its significantly increased risk of severe hepatotoxicity [10]. A significant proportion of residents grossly underestimated the risk of progression from latent tuberculosis infection to active disease in HIV-infected and HIV-uninfected individuals, and did not recognize the importance of infliximab use as a major risk factor in predicting reactivation of tuberculosis [29]. These findings underscore the need to instruct medical residents about the importance of promptly diagnosing and treating latent tuberculosis infection in high-risk populations.

Another common misconception among medical residents was that acid fast smear negativity reliably excludes the diagnosis of pulmonary tuberculosis and precludes transmissibility of infection. This misconception may be based on hospital infection control practice guidelines, which often recommend discontinuation of respiratory isolation for tuberculosis suspect patients after demonstration of sputum smear-negativity [30]. However, an epidemiologic study of tuberculosis cases in San Francisco used DNA fingerprinting to demonstrate that although acid fast-negative culture-positive patients were less infectious than acid fast-positive cases, the former group were still responsible for about 17% of *M. tuberculosis *transmission [31].

Our study has several limitations. The survey was conducted at medical residency training programs in Baltimore, MD, and Philadelphia, PA, cities with a moderate prevalence of tuberculosis; the case rates per 100,000 population were 4.5 and 4.2 for Baltimore and Philadelphia, respectively, in 2004 [32]. Therefore, it is possible that our results may underestimate medical resident knowledge in cities with higher case rates of tuberculosis, such as New York City (11.9/100,000 population), Houston (11.2/100,000 population), or Los Angeles (10.0/100,000 population), or overestimate medical resident knowledge in cities or rural areas with a lower prevalence of tuberculosis. However, our data and those of others [21] suggest that medical resident knowledge does not improve with the number of tuberculosis cases seen. Surveys were completed by only 29% of medical residents at the participating institutions, primarily because of the study design. Our survey was administered during a single scheduled resident conference at each of the four participating institutions in order to maximize the number of survey respondents while minimizing the potential for use of outside sources of information. Therefore, the poor survey response rates in fact reflect poor resident conference attendance. On the other hand, survey response rates among conference attendees at each institution were 100%. It is possible that sole inclusion of conference attendees may have introduced inadvertent bias into the subject pool. However, attendance at didactic teaching conferences does not appear to correlate with medical resident knowledge, at least as measured by performance in medical certifying examinations [33]. Finally, we cannot be sure that lack of tuberculosis knowledge leads to poor management of patients. Although trainees may score poorly, they may be quick to consult experts in Infection Control, Infectious Diseases, or Pulmonary Medicine to assist in diagnosis, isolation, and treatment. Resources pertaining to the education of undergraduate and graduate health-related students in the management and control of active and latent tuberculosis infection may be found at the website of the National Tuberculosis Curriculum Consortium.

## Conclusion

The ability of medical residents training at U.S. urban medical centers to diagnose and manage tuberculosis infection has important public health implications. Overall, medical residents appear to have significant knowledge gaps in several aspects of tuberculosis care, including the diagnosis and treatment of latent tuberculosis infection, and the correct interpretation of negative acid-fast smear sputum samples. Since clinical practice paradigms are often ingrained in physicians during their residency training, education of medical residents on guidelines for detection and early management of tuberculosis may be important for future improvements in national tuberculosis control strategies.

## Appendix 1: Medical resident TB knowledge survey

### Assessment of tuberculosis knowledge among primary care residents in urban medical centers

Given the increasing prevalence of tuberculosis (TB) cases in urban areas related to homelessness, incarceration, immigration, and concurrent human immunodeficiency virus (HIV) infection, it is imperative that primary care physicians in urban medical centers are able to recognize and treat this important infection. The purpose of the proposed research is to assess the adequacy of U.S. graduate medical training regarding TB in large urban medical centers, which has not been studied previously.

We are conducting a survey of 1^st^, 2^nd^, and 3^rd ^year resident physicians enrolled in Internal Medicine training programs at Johns Hopkins Hospital, Johns Hopkins Bayview Medical Center, University of Maryland, and University of Pennsylvania. Surveys consist of 20 multiple-choice and true/false questions related to the epidemiology, diagnosis, and management of patients with TB.

Your participation in this study is entirely VOLUNTARY. Therefore, you may choose not to participate at any time. If you choose to participate, you will have 30 minutes to complete the survey, which must be done without the aid of textbooks or other auxiliary materials. Surveys will bear NO IDENTIFYING INFORMATION, other than year of residency training. Upon completion of the study, you will be provided with the study results, including correct responses to each question and pertinent explanations.

### Assessment of tuberculosis knowledge among primary care residents in urban medical centers

#### Please answer the following background questions (check only one)

Post-graduate year (PGY) of training (Intern = PGY 1, etc.):

□ PGY 1 □ PGY 2 □ PGY 3 □ Other

Number of patients with tuberculosis for whom you have directly cared in the last year:

□ None □ 1–2 □ 3–5 □ 5–10 □ More than 10

#### For each of the following case scenarios, please circle the most appropriate answer (circle only one)

1. A 32 year-old asymptomatic woman who recently emigrated from Vietnam has a 12 mm indurated reaction 72 hours after a tuberculin skin test was placed for employment purposes. Her chest X-ray is unremarkable. She has normal liver function tests. She states she received the BCG vaccine at birth and again at age 20. The next course of action is:

a. Four-drug therapy with isoniazid, rifampin, ethambutol, and pyrazinamide for 2 months, followed by 4 months of isoniazid and rifampin

b. Isoniazid with vitamin B6 for 9 months

c. Sputum sample for acid-fast bacilli and cultures

d. No treatment; the positive tuberculin skin test may be explained by the history of BCG vaccination

2. A 29 year-old homeless man presents to your clinic with a 2-month history of fevers, night sweats, and weight loss. He denies cough or hemoptysis. On physical examination you detect cervical lymphadenopathy, and a chest X-ray reveals normal lung fields but enlarged mediastinal lymph nodes. You suspect extrapulmonary tuberculosis. You place a tuberculin skin test. The next course of action is:

a. Sputum sample for acid-fast bacilli and cultures

b. Cervical lymph node biopsy for acid-fast staining and mycobacterial cultures

c. Blood cultures for mycobacteria

d. Serum early secreted antigen-6 (ESAT-6)

3. One of your co-workers is being relocated to Mali for 2 years. He inquires about tuberculosis prophylaxis. You recommend:

a. BCG vaccination

b. Daily isoniazid during his stay

c. Weekly rifampin during his stay

d. The use of N-95 respiratory masks while in crowded public places

e. No specific prophylaxis

4. A 26 year-old asymptomatic man who recently immigrated from El Salvador is found to have a positive tuberculin skin test. The following are acceptable treatments for latent tuberculosis infection EXCEPT:

a. Daily isoniazid for nine months

b. Daily rifampin for 4 months

c. Daily rifampin and pyrazinamide for 2 months

5. A 5-mm reaction after tuberculin skin test placement is considered positive for all of the following cases EXCEPT:

a. 38 year-old woman with HIV

b. 24 year-old husband of a known TB case

c. 67 year-old man with fibrotic changes in the right upper lobe on chest X-ray

d. 52 year-old woman on immunosuppression for renal transplant

e. 32 year-old nurse working at university student health clinic

6. The **lifetime **risk of developing active disease in non-HIV-infected patients with latent tuberculosis infection is:

a. 0.1–0.5%

b. 1–2%

c. 5–10%

d. 25–35%

e. 50–75%

7. The **annual **risk of developing active disease in patients with HIV and latent tuberculosis infection is approximately:

a. 0.1%

b. 1%

c. 10%

d. 25%

e. 50%

8. You have diagnosed a 30 year-old man with pulmonary tuberculosis and have started therapy with a four-drug combination including isoniazid, rifampin, pyrazinamide, and ethambutol. You should advise your patient of the following EXCEPT:

a. To avoid the use of contact lenses

b. To limit the consumption of green leafy vegetables

c. To avoid alcohol consumption

d. To report changes in color vision

e. To report numbness or tingling of the extremities

9. A patient with no prior medical history is diagnosed with pulmonary tuberculosis and you prescribe a daily combination regimen containing isoniazid, rifampin, pyrazinamide, and ethambutol. The patient sees his primary care doctor 3 weeks later for a scheduled office visit, at which time routine chemistry tests reveal a 2-fold elevation of the aspartate aminotransferase (AST) above a normal baseline value. At this point, the patient should:

a. Discontinue isoniazid only

b. Discontinue rifampin only

c. Discontinue rifampin and pyrazinamide

d. Discontinue all anti-TB drugs until AST returns to baseline

e. Continue current regimen and monitor symptoms and transaminases

10. Pyridoxine supplementation (25 mg/day) during treatment of latent tuberculosis infection is recommended for the following patients EXCEPT:

a. 31 year-old pregnant woman

b. 28 year-old man with HIV

c. 74 year-old man with peripheral neuropathy

d. 21 year-old woman with anemia

11. A 67 year-old woman with rheumatoid arthritis and chronic obstructive pulmonary disease presents with a right upper lobe cavitary lesion. Her greatest risk factor for tuberculosis is:

a. Age

b. History of rheumatoid arthritis

c. Prior use of methotrexate

d. Smoking history

e. Prior use of infliximab

#### The following are TRUE or FALSE statements. Please check the most appropriate answer (check only one)

12. Patients who are AFB smear-negative cannot transmit *Mycobacterium tuberculosis*.

□ True □ False

13. Patients with extrapulmonary disease are less likely to transmit *Mycobacterium tuberculosis *than those with pulmonary involvement.

□ True □ False

14. The diagnosis of pulmonary tuberculosis can be reliably excluded on the basis of a negative tuberculin skin test.

□ True □ False

15. The diagnosis of pulmonary tuberculosis can be reliably excluded after 3 sequential sputum samples are negative for acid-fast bacilli (AFB).

□ True □ False

16. The diagnosis of pulmonary tuberculosis can be reliably excluded after a single bronchoscopic alveolar lavage sample is negative for acid-fast bacilli (AFB).

□ True □ False

17. A positive sputum sample using a nucleic acid amplification technique (e.g., the MTB direct test) indicates the diagnosis of tuberculosis.

□ True □ False

18. A negative nucleic acid amplification test (e.g., the MTB direct test) performed on an AFB-negative sputum sample can reliably exclude the diagnosis of tuberculosis.

□ True □ False

19. Of first-line anti-TB agents, rifampin has the greatest potential for drug-drug interactions in patients receiving highly active antiretroviral therapy (HAART).

□ True □ False

20. In HIV-infected patients with CD4 < 100, HAART should be withheld until active tuberculosis has been completely treated.

□ True □ False

## Appendix 2: Survey answer key (percent of all residents responding correctly)

1. B (47%)

2. B (56%)

3. E (81%)

4. C (41%)

5. E (66%)

6. C (45%)

7. C (47%)

8. B (62%)

9. E (70%)

10. D (15%)

11. E (55%)

12. F (72%)

13. T (87%)

14. F (97%)

15. F (28%)

16. F (40%)

17. T (76%)

18. F (66%)

19. T (84%)

20. F (79%)

## Competing interests

The author(s) declare that they have no competing interests.

## Authors' contributions

PK conceived the study and drafted the manuscript. FS performed the statistical analysis and helped to draft the manuscript. RM helped carry out the statistical analysis. VA participated in the study design and helped to draft the manuscript. KP participated in the study design and helped to draft the manuscript. YM participated in the study design and helped to draft the manuscript. JC participated in the study design and helped carry out the statistical analysis. All authors read and approved the final manuscript.

## Pre-publication history

The pre-publication history for this paper can be accessed here:


